# The Value of a Synthetic Model-based Training Lab to Increase Proficiency with Endoscopic Approaches to the Spine

**DOI:** 10.7759/cureus.7330

**Published:** 2020-03-19

**Authors:** Gregory Basil, G Damian Brusko, Jordan Brooks, Michael Y Wang

**Affiliations:** 1 Neurological Surgery, University of Miami Miller School of Medicine, Miami, USA; 2 Neurological Surgery, University of Miami, Miami, USA

**Keywords:** endoscopic spine surgery, transforaminal, neurosurgical training, spine surgery

## Abstract

Introduction: The learning curve associated with endoscopic approaches to the spine is well established. In this study, we present our endoscopic training methodology and discuss the concepts and rationale of laboratory training as it relates to improving comfort and skill with endoscopic techniques.

Materials and Methods: A three-part endoscopic training laboratory for neurosurgical trainees and attendings was organized at the University of Miami, which included a lecture, instrumentation demonstration, and both synthetic model and cadaveric practice sessions. Participants completed pre- and post-lab surveys gauging their comfort and competency in the transforaminal approach to the lumbar spine.

Results: There were a total of 22 participants, with eight completing the pre-lab survey and 10 completing the post-lab survey. Sixteen participants engaged in the lab practical, with six of these participants performing the transforaminal approach on both the model and the cadaver. An increase in comfort level was demonstrated on the post-lab survey (5.9/10) for the transforaminal approach as compared to the pre-lab survey (2.6/10). Additionally, participants found the training model to be an effective teaching aid for the transforaminal technique (8.8/10).

Conclusions: We believe that our study demonstrates the utility of simulated model-based training for gaining comfort and proficiency with endoscopic approaches to the spine and introduces a safe, cost-effective method of educating practitioners on novel endoscopic approaches.

## Introduction

There has been significant progress in the development of endoscopic techniques to treat spinal pathology over the past few decades [1]. While these surgeries were initially limited to decompressive procedures, endoscopic-assisted fusion surgeries are now gaining increasing popularity [2-9]. These approaches offer the same benefit as traditional open approaches with reduced blood loss, decreased postoperative pain, and earlier discharge [2,10,11]. Nevertheless, the steep learning curve associated with these procedures has been well outlined in the literature and is likely one of the greatest barriers to more widespread acceptance of these techniques.

There have been various solutions for overcoming this learning curve including enhanced academic conferences, laboratory training, and early supervision [12-15]. Laboratory training for these approaches can take various forms, including traditional lectures, cadaveric sessions, and training model-based practice.

In this paper, we present our endoscopic training methodology for a laboratory-based session and discuss the concepts and rationale of model-based training as it relates to improving surgeon comfort and skill levels with endoscopic techniques.

## Materials and methods

An endoscopic training laboratory was organized at the University of Miami surgical lab center for neurosurgical residents, fellows, and attendings. This lab consisted of a three-part course designed to introduce the concepts of endoscopic spine surgery, familiarize participants with the techniques and instrumentation, and improve comfort and skill levels with the procedure.

First, a senior neurosurgical resident with extensive endoscopic training presented a formal lecture highlighting the key anatomic landmarks for an endoscopic transforaminal approach, with particular attention focusing on Kambin’s triangle. Participants were encouraged to utilize several lumbar spine models during the lecture period in order to enhance their understanding of the spatial relationships between the boundaries of Kambin’s triangle prior to practicing the approach. The appropriate surgical technique for performing an endoscopic transforaminal approach to the lumbar spine was also reviewed in detail.

Next, participants were brought to the lab for a brief introduction to the various instruments used in this procedure. For the purposes of this lab, we employed the Spinendos (München, Germany) endoscope and associated equipment. An industry representative was available in the lab to demonstrate proper use of each instrument. A senior faculty member experienced in endoscopic techniques demonstrated the transforaminal approach on a spine training model. For this lab, we utilized the MISSTRAINER, produced by Creaplast (Verton, France).

Following the demonstration, participants attempted a transforaminal approach on the training model. There was no formal time or attempts limit during the training model session, such that each participant practiced until he or she felt comfortable with needle localization of the neural foramen. Following successful foraminal localization, the spine models were replaced with cadaveric specimens, and the same exercise was repeated.

**Figure 1:**
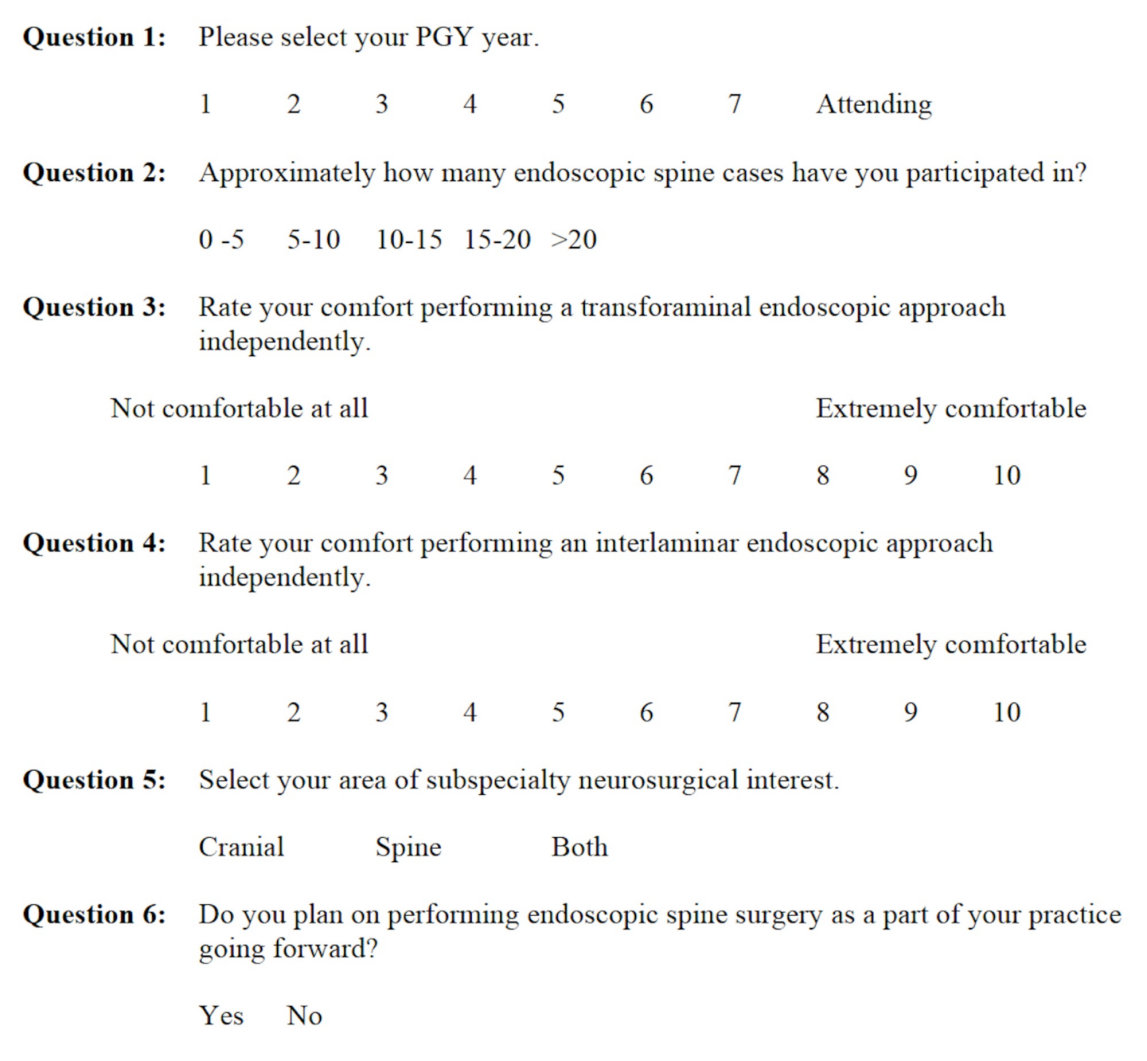
Pre-lab survey. PGY: postgraduate year.

**Figure 2:**
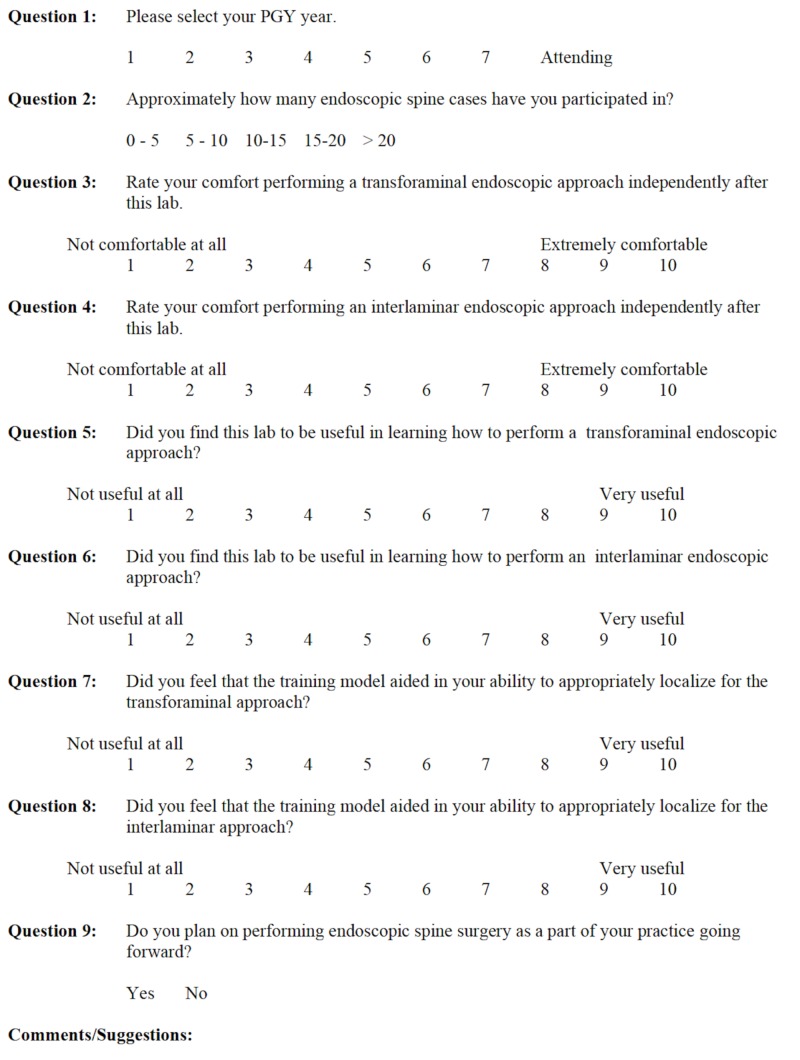
Post-lab survey. PGY: postgraduate year.

In order to simulate as realistic a surgical experience as possible, an experienced X-ray technician performed fluoroscopy throughout the lab session, and the surgical scrub technician used in all endoscopic cases assisted the participants as well. Additionally, participants were asked to voluntarily complete a pre- and post-lab survey (Figures 1, 2) designed to gauge their subjective pre- and post-lab knowledge and technical comfort level.

## Results

There were a total of 22 participants, with two attendings and one fellow, during the lecture period (Table 1). Sixteen of these participants engaged in the lab practical session, with six performing the transforaminal approach on both the model and the cadaver.

**Table 1:** Baseline demographic characteristic of participants who completed the endoscope spinal training lab. N: number of participants; SD: standard deviation; mSV: millisieverts.

Variable	Participants
	n=15, trials=22
Postgraduate year, N (%)	
Postgraduate year 1	2 (13.3%)
Postgraduate year 2	1 (6.7%)
Postgraduate year 3	1 (6.7%)
Postgraduate year 4	3 (20%)
Postgraduate year 5	2 (13.3%)
Postgraduate year 6	3 (20%)
Postgraduate year 7	1 (6.7%)
Spine fellow	1 (6.7%)
Attending physician	2 (13.3%)
Training type, N (%)	
Training model	11 (50%)
Cadaver	11 (50%)
Surgical approach, N (%)	
Transforaminal	16 (100%)
Previous experience, N (%)	
No experience	8 (53.3%)
Experience	7 (46.7%)
Anterior posterior X-ray images, mean (SD)	5.6 (3.8)
Lateral X-ray images, mean (SD)	13.9 (6.1)
Total X-ray images, mean (SD)	19.5 (7.9)
Time to reach surgical target, seconds, mean (SD)	329.7 (137.7)
Disk space entered, N (%)	
No	13 (59.1%)
Yes	8 (36.4%)
Total radiation dosage, mSV, mean (SD)	29.3 (11.9)

Eight participants completed the pre-lab survey and 10 completed the post-lab survey. Of the eight participants who completed the pre-lab survey, four indicated spine, two indicated cranial, and two indicated “both” as their subspecialty interest. Six participants affirmed a future interest in employing endoscopic procedures as part of their practice. The average comfort level was assessed with the pre-lab survey on a scale from 1 (not comfortable at all) to 10 (extremely comfortable) for general fluoroscopic localization (5.9/10) and the transforaminal approach (2.6/10) to the lumbar spine. An increase in comfort level was demonstrated on the post-lab survey, as average comfort increased to 5.9/10 for the transforaminal approach (Table 2).

**Table 2:** Self-reported physician comfort level pre-training compared to post-training for endoscopic approach. Measured using a 10-point Likert scale with 1 being the least comfortable and 10 being the most comfortable. SD: standard deviation.

Characteristic	Pre-training	Post-training	P-value
	n=8	n=10	
Comfort with transforaminal approach, mean (SD)	2.6 (2.3)	5.9 (1.7)	0.0031

Course participants rated the usefulness of cadaveric practice in teaching the transforaminal approach as 9/10 on average. Similarly, participants found the training model to be an effective teaching aid for the transforaminal technique (8.8/10). Complete results from the pre- and post-lab surveys can be found in Tables 3, 4, respectively.

**Table 3:** Pre-lab survey responses. SD: standard deviation.

Variable/question	Participants
	n=8
Postgraduate year, N (%)	
Postgraduate year 1	2 (25%)
Postgraduate year 2	0 (0%)
Postgraduate year 3	1 (12.5%)
Postgraduate year 4	1(12.5%)
Postgraduate year 5	1 (12.5%)
Postgraduate year 6	2 (25%)
Postgraduate year 7	1 (12.5%)
Previous endoscopic spine cases, N (%)	
0-5 cases	7 (87.5%)
6-10 cases	-
11-15 cases	1 (12.5%)
16-20 cases	-
>20 cases	-
Subset neurosurgical interest, N (%)	
Cranial surgery	2 (25%)
Spinal surgery	4 (50%)
Both	2 (25%)
Plans to practice endoscope surgery in future, N (%)	
No	2 (25%)
Yes	6 (75%)
Comfort level with using fluoroscopy for lumbar spine surgery, mean (SD)	5.8 (3.5)
Comfort level with transforaminal approach independently, mean (SD)	2.6 (2.3)

**Table 4:** Post-lab survey responses. SD: standard deviation.

Variable/question	Participants
	n=10
Postgraduate year, N (%)	
Postgraduate year 1	1 (10%)
Postgraduate year 2	-
Postgraduate year 3	-
Postgraduate year 4	1(10%)
Postgraduate year 5	4 (40%)
Postgraduate year 6	1 (10%)
Postgraduate year 7	1 (10%)
Previous endoscopic spine cases, N (%)	
0-5 cases	8 (80%)
6-10 cases	-
11-15 cases	-
16-20 cases	2 (20%)
>20 cases	-
Plans to practice endoscope surgery in future, N (%)	
No	3 (30%)
Yes	7 (70%)
Comfort level with transforaminal approach independently, mean (SD)	5.9 (1.7)
Lab usefulness in learning transforaminal approach, mean (SD)	9 (1.0)
Lab usefulness in localizing for the transforaminal approach, mean (SD)	8.8 (0.9)

## Discussion

The learning curve associated with endoscopic approaches to the spine is a known challenge to more widespread adoption of these techniques [12,13,16,17]. Indeed, there have been several studies that show a steep decline in re-operations as the number of cases performed by the surgeon increases [2,3,12,18,19]. While the relatively rapid acquisition of proficiency in this technique is encouraging, it still poses a problem for the inexperienced practitioner. Needless to say, both the patient and surgeon would be well served if these techniques were refined in a practice setting, rather than in the operating room.

This concept of laboratory and simulated training is not new, nor is it novel to endoscopic spine surgery [20-23]. A national survey of neurosurgery residency programs demonstrated that 95.4% of educators believed laboratory training was integral to training, yet only 50.8% of surveyed training sessions focused on endoscopy [23]. Yadav et al. described a number of pitfalls encountered in endoscopic spine surgery based on experiences of over 1,000 cases and discussed the importance of training mediums available to a young neurosurgeon in order to gain initial endoscopic proficiency [20]. Nevertheless, we were unable to find any literature that attempted to directly address or quantify the benefits of this approach to endoscopic spine surgery.

In the authors’ opinion, one of the more difficult aspects to endoscopic spine surgery is the use of fluoroscopy for localization. While fluoroscopic localization is commonly used in any general spine practice, the level of difficulty and importance of precise fluoroscopic localization is increased with endoscopic surgery. This is especially true for the transforaminal approach where the endoscope is maneuvered into a very small opening and in close proximity to critical neural elements. Therefore, we focused the efforts of our study on gauging proficiency and comfort with this aspect of the procedure.

There was a statistically significant improvement in subjective surgeon comfort in performing the transforaminal approaches after our laboratory session. However, there was no statistically significant difference in the usefulness of the cadaver or synthetic model in terms of their efficacy as a training aid. We believe that this demonstrates the utility of either surgical training medium as a beneficial tool. Cadaveric specimens may limit the widespread adoption of similar training experiences due to their expense. Thus, it appears that more cost-effective training aids, such as synthetic spine models, may provide equal benefit in terms of improved comfort and skill levels, enabling greater ease in organizing training sessions.

There are undoubtedly shortcomings of this study. First and foremost is the small number of participants who performed the transforaminal approach on both the model and the cadaver. This was largely due to time constraints of residents and faculty, and the time-consuming nature of this procedure (especially in practitioners less familiar with this approach). However, while we fully acknowledge the small sample size of the study, we do believe that there are still meaningful takeaways, namely that trainees found the synthetic model-based tool to be a useful means of learning endoscopic approaches to the spine. We also fully recognize the subjective nature of the surveys conducted.

However, rather than using these data to suggest that all participants are fully proficient in endoscopic spine surgery after a single lab, or to suggest that we have definitely proven the value of a synthetic training model for endoscopic approaches to the spine, we do believe that our results suggest the need for further research in this critically important arena. Even with the small number of total participants, we do believe that it indicates a definite progression in knowledge and procedural competence of the lab participants. Furthermore, this study did not quantitatively assess technical parameters, such as localization time or number of fluoroscopic images taken. Measurement of these variables would likely have enhanced our conceptual argument that training models improved technical skill over time and future studies on similar training sessions should explore this relationship further. This study also did not address proficiency or comfort with the remaining components of the endoscopic transforaminal procedure such as complete discectomy and foraminotomy. Although these components were outside the scope of this study, we do believe that they would also be amenable to a simulated model-based training. Finally, while the training models are not perfect simulations, they offer relatively affordable and easy access to ongoing training which we believe will be invaluable to a new practitioner.

## Conclusions

We believe that our study demonstrates the utility of simulated model-based training for gaining comfort and proficiency with endoscopic approaches to the spine. While further research is needed to fully quantify the benefits of these techniques and apply them to the all components of a full endoscopic decompression, we believe that this paper demonstrates a safe, cost-effective method of educating practitioners on novel endoscopic approaches.
